# Secondary aneurysmal bone cyst arising from polyostotic craniofacial fibrous dysplasia

**DOI:** 10.1093/bjrcr/uaaf047

**Published:** 2025-09-09

**Authors:** Niket Patel, Kiran Kumar Sailagundla, Essaki Rajulu, Shanmuga Jayanthan, Ganesh Rajagopal, Harpreet Singh Grewal

**Affiliations:** Department of Microbiology and Immunology, Drexel University College of Medicine, Philadelphia, PA 19129, United States; Department of Radiology, Ascension Sacred Heart Hospital, Pensacola, FL 32504, United States; Department of Radiology, Meenakshi Mission Hospital and Research Centre, Madurai 625107, Tamil Nadu, India; Department of Radiology, Meenakshi Hospital, Thanjavur 625107, Tamil Nadu, India; Department of Radiology, Meenakshi Mission Hospital and Research Centre, Madurai 625107, Tamil Nadu, India; Department of Radiology, Florida State University College of Medicine, Pensacola, FL 32514, United States

**Keywords:** ground glass matrix, expansile bony lesion, blood-fluid level, craniofacial fibrous dysplasia, aneurysmal bone cysts

## Abstract

Fibrous dysplasia (FD) is a benign condition affecting osteoblasts, which fail to undergo proper differentiation and maturation, resulting in the replacement of normal osteoid matrix with ground glass fibrous tissue. Aneurysmal bone cyst (ABC) is a benign, expansile, lytic lesion characterized by multiple blood-filled cystic cavities containing haemorrhagic products at varying stages. Secondary ABC arising from craniofacial FD is extremely rare. To date, only 10 cases have been reported in the literature. This report highlights the clinical presentation, imaging findings, and histopathological confirmation of a secondary ABC in a patient with polyostotic craniofacial FD.

## Introduction

Fibrous dysplasia (FD) is a benign condition affecting osteoblasts that fail to undergo proper differentiation and maturation, resulting in the replacement of normal bone with ground glass fibrous tissue.^1^ An aneurysmal bone cyst (ABC) is a benign, expansile, lytic lesion characterized by numerous blood-filled cystic cavities containing haemorrhagic products.^2^ Secondary ABCs arising from craniofacial FD are extremely rare. Here, we report a case involving FD of the left frontal bone, extending into the facial bones, skull base, mandible, and bilateral optic canals, with a secondary ABC arising from the left frontal bone. To date, only 10 cases have been reported in the literature.

## Clinical presentation

A 24-year-old female presented with complaints of swelling in the left frontal region of the scalp and visual disturbances lasting 2 months. She reported intermittent headaches and had a history of facial deformity spanning several years. On clinical examination, the swelling was cystic and non-tender. The patient was referred to the radiology department for a CT scan. The resection was performed due to progressive visual disturbance and concern for compressive effects from the lesion expansion. Specifically, the lesion’s proximity to the bilateral optic canals raised the possibility of increasing pressure on the optic nerves, which could result in permanent vision loss if not promptly addressed. Additionally, the enlarging mass posed a potential risk of cosmetic deformity and further craniofacial complications if left untreated.

## Differential diagnosis

- Fibrous dysplasia
- Paget’s disease
- Sclerotic metastases
- Aneurysmal bone cyst
- Telangiectatic osteosarcoma
- Giant cell tumour

## Imaging findings

**Figure 1.**
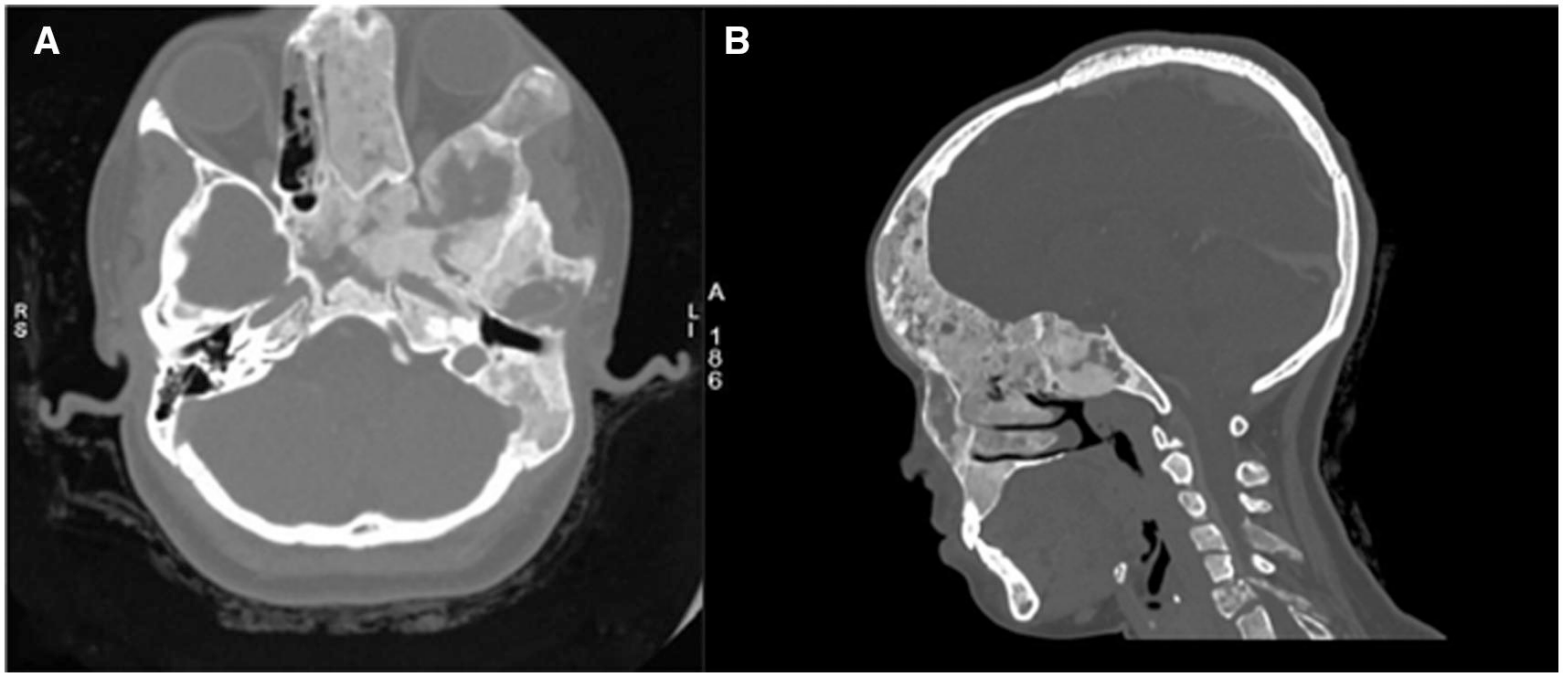
(A) CT cranium axial bone window showing bone expansion with ground glass matrix in left ethmoid sinus, sphenoid bone with narrowing of left optic canal and petrous and mastoid portion of left temporal bone. (B) CT cranium with cervical spine sagittal plane bone window showing ground glass matrix in left frontal bone, maxilla, C4 spinous process, and C6 cervical vertebral body.

**Figure 2.**
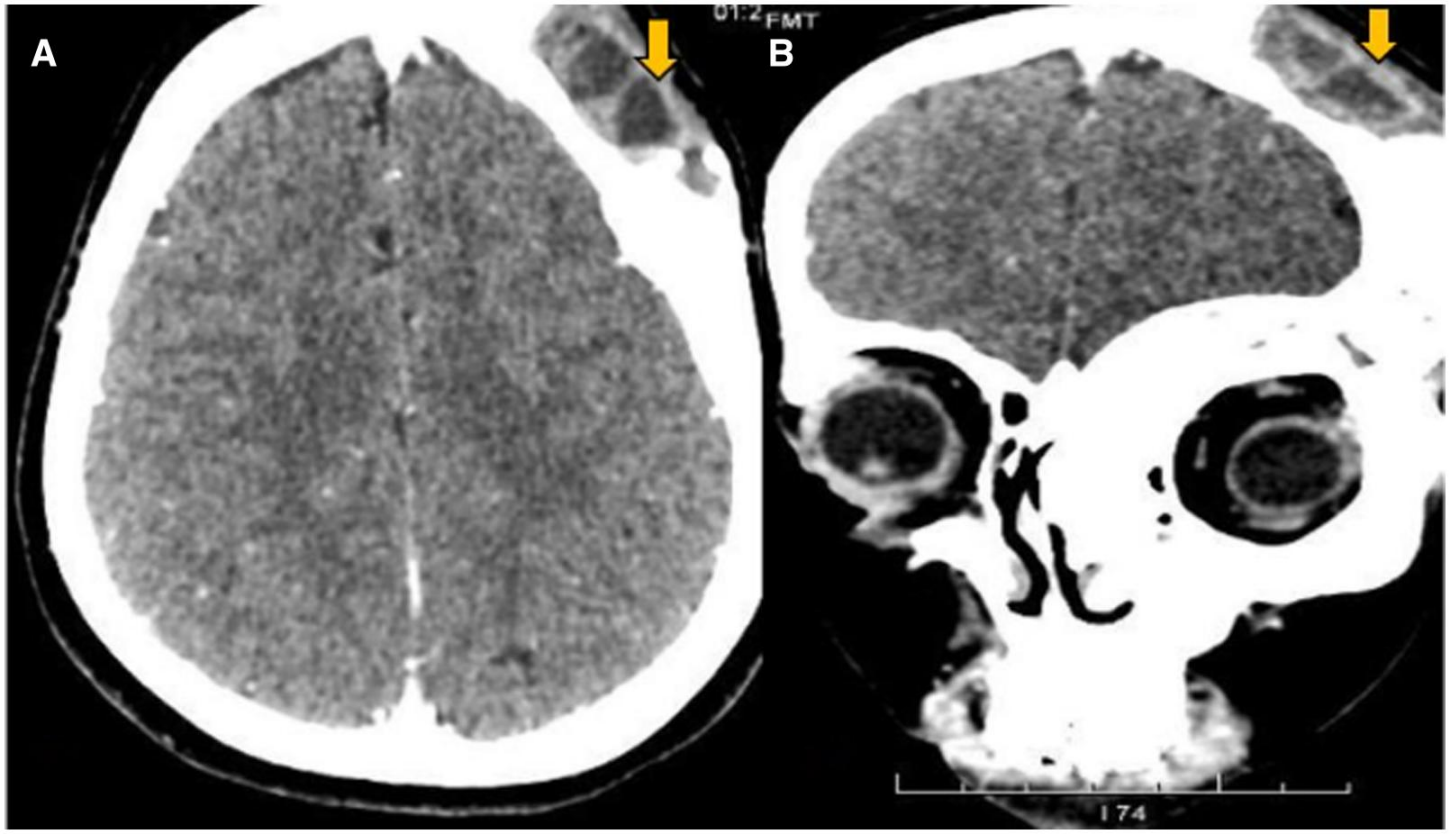
(A) CT brain axial plane soft tissue window showing focal soft tissue and cystic component seen in left frontal bone with osteolytic lesion and blood-fluid level. (B) CT brain coronal plane soft tissue window showing focal soft tissue and cystic component seen in left frontal bone with osteolytic lesion and blood-fluid level.

CT imaging of the cranium and facial bones revealed bone expansion with a characteristic ground glass matrix involving the left frontal and temporal bones, ethmoid sinus, and sphenoid bone. There was also narrowing of the bilateral optic canals, along with involvement of the maxilla, mandible, C4 spinous process, and C6 cervical vertebral body. These findings were consistent with polyostotic FD (Figure 1A and B). Additionally, a focal soft tissue lesion with cystic components and blood-fluid levels was identified in the left frontal bone, suggesting a secondary ABC in pre-existing FD (Figure 2A and B).

## Treatment, outcome, follow-up

**Figure 3.**
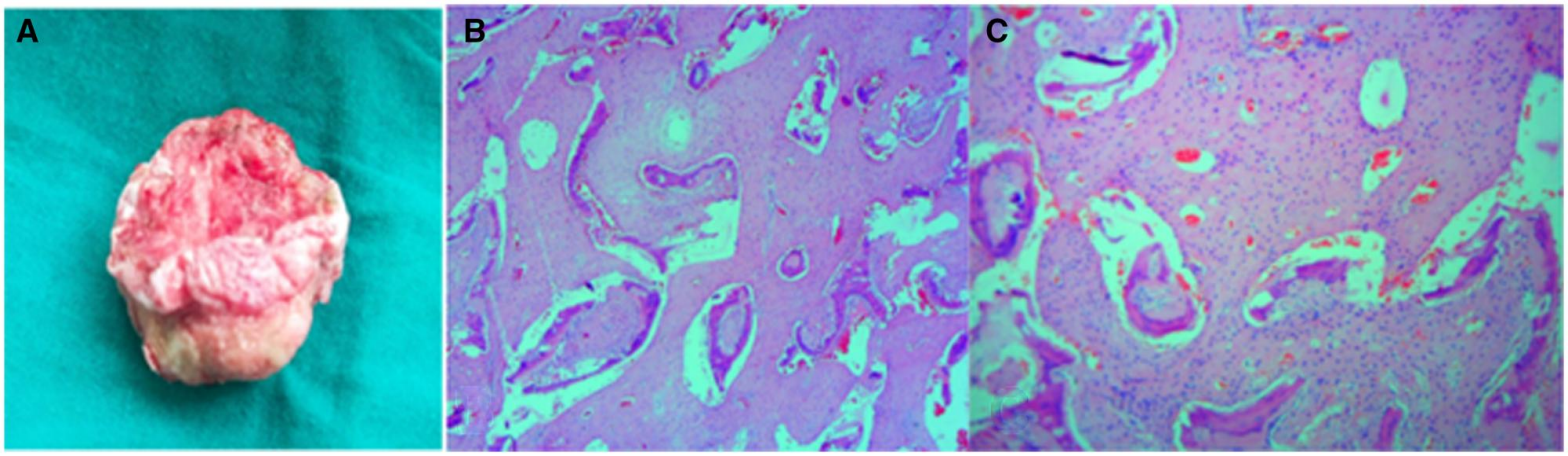
(A) Photograph of the excised specimen. (B) Histological examination of resected lesion with haematoxylin and eosin staining showing curvilinear trabeculae of woven bone within proliferative fibroblastic and vascularized stroma—consistent with fibrous dysplasia. Cystic structure with haemorrhage, reactive woven bone formation and dense inflammatory infiltrates—aneurysmal bone cyst. (C) Curvilinear bony trabeculae with fibrous stroma in between.

The patient underwent surgical excision of the lesion (Figure 3A). Histopathological examination of the resected lesion with haematoxylin and eosin staining revealed curvilinear trabeculae of woven bone embedded in a proliferative, fibroblastic, and vascularized stroma, consistent with FD. The cystic component exhibited haemorrhage, reactive woven bone formation, and dense inflammatory infiltrates, confirming the diagnosis of a secondary ABC (Figure 3B). Microscopic analysis demonstrated trabeculae surrounded by fibrous stroma without any evidence of malignancy (Figure 3C).

The diagnosis of a secondary ABC was made post-operatively based on histopathology. However, the imaging suspicion of an ABC was raised pre-operatively based on the identification of blood-fluid levels within a known FD lesion.

The patient was monitored postoperatively for 1 year. No recurrence was observed at the surgical site, and the other lesions remained static without any signs of secondary changes.

## Discussion

An ABC is a multicameral, metaphyseal, fluid-filled lesion, believed to result from a disturbance of the blood vessels in the affected bone.^3^ It is a benign, osteolytic, expansile bony lesion.^4^ The name refers to its characteristic appearance of expansile, blood-filled cavities, with histological features including haemorrhage, dense inflammatory infiltrates, and giant cells. Although ABCs are benign, they may be active and aggressive in nature. Aneurysmal bone cysts are classified as either primary or secondary. Primary ABCs typically present in isolation, particularly in children and adolescents. They are more common, accounting for approximately 65%-76% of all ABCs, and are frequently found in long bones, flat bones, and the spinal column, with a median age of diagnosis around 11 years. In contrast, secondary ABCs occur in association with other osseous lesions such as giant cell tumours, osteoblastomas, chondroblastomas, osteosarcomas, non-ossifying fibromas, FD, and chondromyxoid fibromas.^5^^,^^6^ These lesions are more often seen in older patients, with a mean age of 32 years in spinal cases, and represent approximately 30%-35% of all ABCs. Secondary ABCs also tend to recur more frequently and may necessitate more aggressive treatment. They are most commonly located eccentrically in the metaphysis of long bones near unfused physeal plates, while craniofacial bones are uncommon sites of involvement.^4^ Within the craniofacial skeleton specifically, secondary ABCs associated with FD are exceedingly rare, with fewer than a dozen cases documented in the literature, underscoring the unique nature of the present case.^6^

The pathogenesis of ABCs is not fully understood, but the most widely accepted theory suggests that they arise due to increased venous pressure and the consequent rupture of local vascular networks. Secondary ABCs may develop in pre-existing bone lesions due to haemodynamic changes caused by abnormal bone.^7^ Fibrous dysplasia can serve as a precursor for secondary ABC, particularly when the lesion exhibits rapid enlargement. Patients typically present with a soft, painful, and rapidly enlarging mass in the context of known FD, warranting curative resection of the ABC and adjacent FD tissue.^7^

Fibrous dysplasia is a benign lesion characterized by intramedullary fibro-osseous proliferation due to impaired osteogenesis. It may present in either monostotic or polyostotic forms. Histologically, FD exhibits curvilinear trabeculae of woven bone within a fibroblastic and vascularized stroma. The lesion can alter normal vascular development within the osseous matrix, leading to haemodynamic changes that may promote the formation of cystic spaces containing blood of varying densities. This process can result in the development of secondary ABCs, a theory postulated by Tresserra et al.^8^

Fibrous dysplasia is a rare, non-inherited bone disorder that arises sporadically due to post-zygotic activating mutations in the GNAS gene, specifically affecting the α-subunit of the stimulatory G protein (Gsα). This leads to constitutive activation of adenylate cyclase and elevated cyclic AMP levels, resulting in abnormal bone development. Fibrous dysplasia does not undergo vertical transmission, and its lesions do not spontaneously resolve. Morphologically, normal bone is replaced with fibrous tissue and immature woven bone. The monostotic form—involving a single bone—does not progress to polyostotic FD or McCune-Albright syndrome (MAS).^9^

Complications in FD includes bone deformities (eg, bowing of limbs), pathological fractures, pain and functional impairment, craniofacial involvement leading to facial asymmetry, or vision/hearing problems and transformation to malignancy (eg, osteosarcoma). It is often associated with MAS when accompanied by endocrine abnormalities and skin pigmentation. Patients with Mazabraud syndrome have FD lesions and myxomas, typically located in the vicinity of the bone lesions.^9^

On imaging, ABCs typically appear as expansile, thin-walled, lytic lesions with characteristic blood-fluid levels seen on both CT and MRI. These levels can also be present in other haemorrhagic osseous lesions.^10^ Fibrous dysplasia, in contrast, is identified on CT by well-defined ground glass opacities, which may be homogeneously sclerotic. Blood-fluid levels may be observed in FD either due to sarcomatous transformation, simple cystic degeneration, or the formation of secondary ABCs.^11^

The key differentiator confirming the diagnosis of a secondary ABC in this case was the histopathological presence of cystic haemorrhagic areas within a pre-existing FD lesion, without evidence of a primary lesion.

Although Paget’s disease and sclerotic metastases are considered in the radiological differential for FD, they are unlikely in a 24-year-old patient and were included for completeness based on imaging appearance. Giant cell tumours are typically solitary lucent lesions located eccentrically in the epiphysis of long bones, usually appearing after the fusion of the growth plate. Telangiectatic osteosarcomas, by contrast, are characterized by cortical destruction, a wide zone of transition, and osteoid matrix calcification.

Although sarcomatous dedifferentiation of FD is rare, it is more commonly observed in the polyostotic form. Therefore, when blood-fluid levels are identified, secondary ABCs should be considered in the differential diagnosis to ensure appropriate treatment. Management strategies differ significantly between benign and malignant conditions. Secondary ABCs in FD typically require simple excision with wide curettage, whereas malignant transformation necessitates adjuvant chemotherapy or radiotherapy.^12^

## Conclusion

Secondary ABCs arising from craniofacial polyostotic FD are rare but should be considered when characteristic blood-fluid levels are seen on imaging. Accurate diagnosis through imaging and histopathology is crucial to differentiate benign from malignant conditions. Timely surgical intervention with excision and curettage can effectively prevent recurrence and complications.

Learning points

- Secondary aneurysmal bone cysts (ABCs) arising from craniofacial fibrous dysplasia (FD) are rare but can be differentiated from malignant transformations through imaging and histopathology.
- Ground glass matrix on CT imaging is a hallmark feature of FD, while blood-fluid levels indicate ABC formation.
- Fibrous dysplasia should be distinguished from other conditions like Paget’s disease and sclerotic metastases using characteristic imaging features.
- Understanding the imaging presentation of secondary ABCs is critical to ensuring appropriate treatment, as malignant transformation requires adjuvant therapy.

## Learning points

- Secondary aneurysmal bone cysts (ABCs) arising from craniofacial fibrous dysplasia (FD) are rare but can be differentiated from malignant transformations through imaging and histopathology.
- Ground glass matrix on CT imaging is a hallmark feature of FD, while blood-fluid levels indicate ABC formation.
- Fibrous dysplasia should be distinguished from other conditions like Paget’s disease and sclerotic metastases using characteristic imaging features.
- Understanding the imaging presentation of secondary ABCs is critical to ensuring appropriate treatment, as malignant transformation requires adjuvant therapy.

## Informed consent

Written informed consent was obtained from the patient for the publication of this case report and associated images. All reasonable measures were taken to ensure patient confidentiality. No identifiable patient information has been disclosed in this manuscript.
